# Developmental Neurobiology of the Rat Attachment System and Its Modulation by Stress

**DOI:** 10.3390/bs2020079

**Published:** 2012-05-18

**Authors:** Reto Bisaz, Regina M. Sullivan

**Affiliations:** 1 Emotional Brain Institute, Nathan Kline Institute, 140 Old Orangeburg Road, Orangeburg, New York, NY 10962, USA; 2 Child & Adolescent Psychiatry, New York University School of Medicine, 215 Lexington Avenue, New York, NY 10016, USA; 3 Center for Neural Science, New York University, 4 Washington Place, New York, NY 10003, USA

**Keywords:** stress, trauma, rat, pup, attachment, maternal odor, fear, amygdala, corticosterone, norepinephrine, locus coeruleus

## Abstract

Stress is a powerful modulator of brain structure and function. While stress is beneficial for survival, inappropriate stress dramatically increases the risk of physical and mental health problems, particularly when experienced during early developmental periods. Here we focus on the neurobiology of the infant rat’s odor learning system that enables neonates to learn and approach the maternal odor and describe the unique role of the stress hormone corticosterone in modulating this odor approach learning across development. During the first nine postnatal days, this odor approach learning of infant rats is supported by a wide range of sensory stimuli and ensures attachment to the mother’s odor, even when interactions with her are occasionally associated with pain. With maturation and the emergence of a stress- or pain-induced corticosterone response, this odor approach learning terminates and a more adult-like amygdala-dependent fear/avoidance learning emerges. Strikingly, the odor approach and attenuated fear learning of older pups can be re-established by the presence of the mother, due to her ability to suppress her pups’ corticosterone release and amygdala activity. This suggests that developmental changes in stress responsiveness and the stimuli that produce a stress response might be critically involved in optimally adapting the pup’s attachment system to its respective ecological niche.

## 1. Introduction

Stress is an adaptive reaction that helps the organism to cope and respond to changing and challenging environmental situations. Such stressful situations, in which homeostasis is challenged physically and psychologically, initiate the activation of the sympathetic-adrenomedullary system and the hypothalamic-pitutary-adrenal (HPA) axis, the latter resulting in increased glucocorticoid blood levels [1]. While the activation of the stress system is necessary for an individual’s survival, frequent or prolonged stress can exert negative effects on most physiological systems [2]. Growing evidence also indicate that inappropriate stress responses might be associated with the development and exacerbation of physiological alterations and neuropsychiatric disorders [3,4,5,6]. In particular, frequent and/or extensive stress during early life has been linked with a dramatic increase in the risk for later development of psychiatric illness and behavioral dysfunction [4,7,8,9]. Although there has been progress in determining the neurobiological consequences of early life stress in humans, most of our current understanding of these effects is derived from animal models in which the central nervous system and behavioral patterns can be explored in response to discrete environmental events. These studies have provided evidence that disturbances of the early social environment, particularly of the mother-infant relationship, can exert profound and lasting impacts on brain development, gene expression, neuroendocrine systems regulating stress responsivity, anxiety and affective disorders [10]. 

Since the altricial rat is born hairless and with only limited sensory and motor functions, it must depend on the mother to receive warmth, nutrition and protection. Therefore, newborns must display an immediate and persistent motivation to identify and approach their mother and attach to her in order to ensure survival. Similar attachment processes of neonates occur in many species and involve the formation of a mutual bond between the infant and the caregiver that ensures the maintenance of physical proximity to the caregiver, which is necessary to signal needs and receive parental care. While a broad knowledge of attachment processes have been established in various mammalian species over the last half century, the laboratory rat has been particularly useful to unravel the neurobiology that underlies certain forms of attachment to the caregiver during early development. Although in psychology the term attachment generally refers to the complex cognitive process of human infants that result in a highly specific attachment process to the primary caregiver [11,12,13], neonatal rat pups show specialized behaviors that can be characterized as specific forms of attachment or bonding. Such attachment behaviors include the strong and persistent proximity seeking behaviors towards the mother’s ventrum (a potent source of maternal odor’s), as well as grasping the nipple and nursing. 

In order to describe stress and attachment interactions, it is important to consider the ecological niche of the infant. Studies in rats have documented that during the earliest postnatal days the mother’s sensory stimulation, such as licking, grooming and nursing critically regulate the activity of the HPA axis in the pups. During this developmental period, when the infant rat is confined to the nest and frequently nursed by the mother, circulating corticosterone (CORT) levels remain low at basal levels and fail to increase in response to most transient stressors. This period, termed the “stress hyporesponsive period” (SHRP) appears to not only protect the developing brain from the deleterious effects of elevated CORT and other neurochemicals associated with the mammalian stress response but -as will be discussed below- is also necessary for the strong and persistent odor approach learning that supports attachment to the mother, even when the interactions with the mother are sometimes associated with pain [14,15]. With maturation and the infant rat’s transition from an altricial to an independent organism, the response to stress and its associated effects undergo important changes that are critically involved in modulating the infant-mother attachment system for an optimal adaptation to the pups’ developmental niche. 

In this review, we will focus on the infant rats and review the current literature on the unique role of stress and CORT in modulating the infant’s attachment system across development. To this end we will first describe the neural system by which maternal odors are able to exert a strong attractive effect in neonatal rat pups, resulting in a strong and persistence odor approach and attachment, regardless of the quality of the care she provides. We will then explore the unique role of CORT in controlling developmental changes of the attachment system, as the newborn rat makes the rapid transition from altricial to an independent organism within just three weeks. Finally, we will provide evidence of how the attachment system is altered by CORT in conditions where the mother is maltreating her pups and how such abusive attachments critically influence the developmental trajectories of the infant, resulting in life-long consequences on brain and behavior. Due to the recent progress made in understanding the neuronal basis of the early social attachment system in rodents and the unique role of stress and CORT in modulating this finely tuned attachment system across development, we believe that it is time for a new and comprehensive review that incorporates recent discoveries with earlier findings.

## 2. Odor Attachment Learning of the Infant Rat

In contrast to other mammalian species, the rat’s auditory and visual systems are not functional until around two weeks after birth. Therefore the newborn pups rely solely on their olfactory systems to initially approach their mother, attach to her nipples and remain in close physical contact with her [16,17,18]. Even though nipple attachment behaviors also rely on perioral somatosensory cues, the presence of maternal odors are necessary to induce nipple localization and suckling behaviors in newborn rat pups, as washing or chemical lavage of the nipple region of lactating females results in increased latency of the pups to find, attach and suckle the nipples [18,19]. Likewise, olfactory bulbectomy or peripheral destruction of the olfactory epithelium with zinc sulfate irrigation significantly diminishes body weight gain and raises mortality rates within the first few days after birth [20,21,22]. Thus, even during the first encounter with the mother, rat pups must readily display a strong behavioral competence for successfully approaching and interacting with the mother in order to survive. 

While pups’ approach and nipple attachment were initially suggested to be guided by pheromones, we now understand that rat pups must learn the mother’s odor [23]. This learning of the maternal odor begins prenatally during the late gestation period, when swallowing emerges and the relatively well-developed gustatory and olfactory systems of the fetus enable the detection of odorants within the amniotic fluid [17,24,25,26,27,28]. The importance of olfactory cues within the amniotic fluid to guide the pup’s directed behavioral response immediately after birth has been demonstrated by placing an artificial odor into the amniotic fluid a few days before birth [17,26,27,29]. This prenatal olfactory experience is retained postnatally and critically supports the pups’ first nipple attachment [30,31]. The prenatal experience with odors seems to also shape the development of the olfactory sensory system’s neural circuitry and has been shown to greatly influence later food preference [32]. Thus, at birth, maternal odors have already been learned and shaped the development of the olfactory system resulting in a persistent maternal odor approach response and nipple attachment behaviors, which are necessary to keep pups warm, protected and well-fed. Additionally, amniotic fluid odors, which surround the nest at birth, may also help to facilitate the newborn’s transition from the intrauterine world into a world filled with new sensory experiences including new odors, textures and temperatures.

The dramatic olfactory learning abilities of rat pups continue postnatally when they are able to rapidly learn to prefer and approach new odors simply by placing a neutral odor on the mother or in the cage during mother-infant interactions [33,34,35]. This early odor attachment learning can also be modeled experimentally outside the nest using classical conditioning in which a novel odor is paired with a sensory stimulus, such as tactile stimulation/stroking, milk or warmth [36,37,38,39,40,41,42]. Importantly, such postnatally acquired odors learned either naturally within the nest or experimentally through conditioning are able to evoke a similar complex sequence of proximity seeking/approach response and nipple attachment behaviors as the natural maternal odor. Since the rat maternal odor is dependent upon her diet, pups must be capable of rapidly relearning her changing odor, in order to maintain a successful interaction with the mother [43,44]. As this infant odor attachment learning is unique in its acquisition, it has been characterized as an “imprinting-like process” that is likely to be central for attachment in slow-developing mammals [45]. Some unique characteristics of infant rat’s odor learning system seem to potentiate the learning of new odors. For instance, while procedures that retard or inhibit associative olfactory learning in adults, such as pre-exposure to an odor before conditioning (*i.e.*, latent inhibition) or temporally unrelated presentations of an odor and reward (*i.e.*, learned irrelevance), have even been shown to enhance odor learning in infant rats [46,47,48,49,50,51,52]. Thus, the prenatal exposure to maternal odors in the amniotic fluid, together with the facilitated postpartum odor learning abilities, enables rat pups to persistently approach the mother and remain in close contact with her, which ultimately underlies the strong mother-infant attachment.

Neonatal rat pups even learn to approach and prefer a novel, artificial odor when it is paired with painful sensory stimulus, such as tail pinches or moderate (0.5 mA) electric shocks. Similarly to odor-stroke conditioning, repeated pairings of an odor with 0.5 mA electric footshock, results in pups approaching it when it is next encountered [53,54,55,56,57]. Notably, such odors that are learned through odor-pain conditioning, acquired similar hedonic values as odors that were paired with a presumably pleasant stimulus (*i.e.*, stroking, milk or warmth) and induce similar and persistent odor approach responses and social behaviors as the natural maternal odor [58]. While this odor attachment learning may seem precarious at first sight, it may have evolved to prevent pups from learning to avoid or inhibit responses towards the mother, as transient painful stimuli are part of typical maternal behaviors as she occasionally handles pups roughly or tramples them, such as when she enters or leaves the nest [14,59,60]. This reduced ability of rat pups to learn an avoidance to odors that are associated with pain might be similarly important in supporting the pup’ attachment to the mother as odors associated with maternal care and nursing and ensures a rapidly and robustly learning of the maternal odors regardless of the quality of maternal care [45,61,62,63,64]. It should be noted that pain-induced odor preference learning of rat pups is neither due to the pups’ inability to detect pain nor to differences in pain threshold [56,65,66,67,68] but, as will be presented below, is dependent upon the unique neurobiological system that prevents and/or attenuates pups’ ability to learn an avoidance to odors associated with painful stimuli. Similar to rats, the ability of neonates to learn a preference for stimuli that are associated with pain, have also been reported in chicks [69,70], infant dogs [71] as well as in human and nonhuman primates [9,72,73,74] and seems to be evolutionary conserved in altricial animals. Considering the importance of proximity seeking behaviors to obtain warmth, nutrition and protection, such an attachment system may greatly increase the chances of infant’s survival through learned approach response towards the caregiver, even if this attachment learning is associated with maltreatment. 

Although learning the maternal odor may reflect the most relevant odor learning during the early postnatal life, since it is directly linked to survival, the neonatal rat pup is also able to learn many other odors and aspects of the early life environment, that result in food and huddling preference [75,76], homing/nest-seeking behaviors [77,78] and thermotaxis [79], to mention just a few. These odor preferences are likely to be acquired similarly as the maternal odor, involving repeated pairings of the odor with sensory stimulation and might also involve similar neurobiological mechanisms, but this hasn’t yet been investigated on the cellular and neural levels. 

## 3. Neural Encoding of the Maternal Odor

Maternal odor learning in rat pups that can occur experimentally, by pairing a neutral odor (*i.e.*, peppermint or citral) with either a pleasant or painful stimulus, or naturalistically within the nest, all produce an increased neuronal activity within the olfactory bulb when these odors are subsequently encountered, as repeatedly assessed by techniques such as [14C]-2-deoxyglucose autoradiography, c-Fos expression, optical imaging, phosphorylation of the cAMP response element-binding protein (CREB) and electrophysiology [35,53,60,80,81,82,83,84,85,86,87,88,89,90,91,92]. These learning-induced changes in neuronal activity of the olfactory bulb that occur in response to postnatally acquired odors are unique to rat pups during the first ten days after birth and have been shown to produce long-lasting consequences on brain and behavior [88,93,94,95].

The involvement of specific neurotransmitters in neonatal odor learning was first demonstrated by Pedersen *et al.* [17], who showed that a systemic injection of amphetamines paired with an hour long presentation of an artificial odor was sufficient to produce behavioral responses that were comparable to those observed in response to natural maternal odors, such as odor approach and nipple attachment. Since then, norepinephrine (NE) has been identified as having an essential role in infant odor learning as well as in the learning-induced changes of olfactory bulb activity [61,91,96,97,98]. In particular, the importance of NE in the olfactory bulb for neonatal olfactory learning has been demonstrated through a series of experiments directly manipulating NE levels in the olfactory bulb. These experiments revealed that infusions of NE into the olfactory bulb simultaneously with the presentation of an artificial odor was sufficient to produce a persistent odor approach response, whereas blocking bulbar NE receptors with the β-adrenergic receptor antagonist propranolol completely prevented pups from learning an odor preference [61,81,91,96,97,98]. The essential role of NE in pup’ odor approach learning was further supported through direct activation of the olfactory bulb’s sole source of NE [61], the locus coeruleus (LC) [99]. The infant LC is uniquely activated in response to a myriad of sensory stimuli, such as stroking tactile stimulation, foot shocks and air puffs [100,101]. Therefore the temporal coincidence of LC induced high levels of bulbar NE, together with the activation of olfactory bulb glomeruli, appears to underlie the neonatal’s ability to learn a new odor. Indeed, NE appears to prolong neural activity of mitral/tufted cells, the primary output neurons of the bulb, which is critical for the occurrence of synaptic plasticity within the olfactory bulb that most likely underlies neonatal olfactory learning [87,91,102,103,104]. 

Additional neurotransmitters have also been reported to influence pups’ odor learning abilities. For instance, selective depletion of serotoninergic (5-HT) neurons in the olfactory bulb during odor-stroke conditioning prevented pups from learning an odor preference [39]. However, infusion of a non-selective 5-HT receptor agonist into the pups’ olfactory bulb during odor presentation failed to produce odor preference learning, but instead facilitated learning in the presence of suboptimal bulbar NE levels [105], indicating a modulatory role of 5-HT in olfactory learning in rat pups. Dopamine (DA) has also been reported to modulate neonatal odor learning through the D1 receptor. Indeed, while systemic injections of a D1 receptor antagonist were able to attenuate (but not block) odor approach following odor-stroke conditioning, D2 receptor blockage remained largely ineffective [42]. The inhibitory neurotransmitter γ-aminobutyric acid (GABA) has also been shown to influence olfactory learning in infant rats, with either GABA_A_- or GABA_B_-receptor antagonist infusion into the olfactory bulb facilitating odor approach learning [103,106]. However, these injected rat pups showed not only a behavioral response to odors paired with a somatosensory stimulus but also to unfamiliar, neutral odors, indicating that blockage of GABAergic transmission in the olfactory bulb might prevent odor-specific learning, perhaps through a disruption of lateral inhibition of activated mitral cells. Finally, opioids [107,108] and oxytocin [109] have also been implicated in modulating odor learning in older pups. Although these studies suggest that various neurotransmitters are able to modify olfactory learning through actions in the olfactory bulb, NE seems to have a unique and essential role in the rat’s odor attachment learning system. 

The NE dependent olfactory learning system described above disappears at around postnatal day (PND) 10, which coincides with changes of neural activity within the LC in response to sensory stimulation. Specifically, while the infant LC shows a prolonged 20 to 30 sec neural response to a 1 sec stroking or electric foot shock, the adult LC shows only a millisecond excitatory response to the same type of stimulations [100,101,110,111]. This developmental difference in neural activity of the LC results in a dramatic reduction of NE release into the olfactory bulb in pups older than 10 days, which prevents the release of sufficient NE to permit the plasticity needed to induce odor approach learning. Indeed, when microdialysis was used to assess NE levels within the olfactory bulb of pups below the age of 10 days, a 2-fold higher NE concentration within the bulb was measured in response to LC or sensory stimulation, as compared to older pups or adults [112,113]. These developmental changes in the LC’s neural response and NE release appears to be due to the functional emergence of the inhibitory α2-noradrenergic autoreceptors that quickly terminate the LC’s excitatory responses following sensory stimulation [114]. Thus, after PND10, NE takes on a modulatory role of learning and memory processes, similar to the ones described in adult rats [115,116,117].

In addition to the olfactory bulb, other brain areas have been identified as mediating pups’ odor learning. The piriform cortex, which plays a central role in processing of olfactory information and is part of the olfactory cortex, has been recently identified to be involved in the infant odor attachment learning. The piriform cortex, which receives monosynaptic input from mitral cells of the olfactory bulb—whose activity are enhanced in response to NE—can be physiologically and anatomically divided into an anterior and a posterior part [118,119]. The activity of the anterior piriform cortex is mainly influenced by inputs from the olfactory bulb and is associated with simple olfactory learning, the posterior part receives excitatory and inhibitory input from various limbic structures, such as the amygdala, the hippocampus and the entorhinal cortex and appears to have an important role in assigning the hedonic value to a learned odor [120,121,122,123,124]. Importantly, while odors learned during early life preferentially engage the anterior piriform cortex, the posterior part does not exhibit learning induced changes until PND10, when the sensitive period for attachment learning ends [53].

The amygdala, which plays a central role in the processing of emotionally arousing stimuli in adult rats [125,126,127,128,129,130,131,132,133,134,135,136,137,138,139], has been shown to not be involved in odor attachment learning in neonatal rat pups, even when the sensory stimuli paired with the odor are painful [56,58,60,140,141]. It should be noted that a lack of amygdala activity has also been found in rat pups that received the odor and sensory stimulus presentation in a non-overlapping/unpaired manner as well as pups that were presented with a novel odor alone, indicating that both novel and/or ambiguous stimuli seem equally not to activate the amygdala at this early developmental period [55,82]. Recent research has explored potential mechanisms of the amygdala’s failure to exhibit plasticity during the sensitive period for attachment learning. While the original hypothesis was that the amygdala was simply too immature to mediate odor avoidance learning, resent findings indicated this was not the case. Indeed, it appears that modified or impaired synaptic plasticity of the cortical inputs to the amygdala (more precisely the basolateral complex) underlies the reduced ability of pups during the sensitive period (*i.e.*, PND ≤ 10) to learn an aversion to odors experienced with painful stimuli [142]. The slow ontogeny of GABA-receptors expression in the amygdala together with finding that the abnormal synaptic plasticity in the amygdala of neonatal pups can be mimicked in older pups through blockage of GABA_A_-receptors, indicate that reduced maturation of GABAergic functions might to some extent be responsible for the reduced plasticity of the amygdala during the early postnatal period [142,143,144]. In support of this hypothesis is the fact that CORT, which is known to modulate GABAergic inhibition and principal cell excitability in the adult amygdala [145], also modulates odor aversion learning of pups during the sensitive period. More precisely, odor aversion learning of pups during the sensitive period can be induced pharmacologically by increasing pups’ CORT levels within the amygdala during odor-shock conditioning, indicating an important role of CORT and stress in modulating amygdala activity and odor aversion and/or fear learning [82,146].

## 4. The Role of Corticosterone in the Termination of the Sensitive Period for Attachment Learning

The rat pups’ ability to learn a new maternal odor, either through pairings with a presumably pleasant stimulus or through odor-0.5 mA shock conditioning, ends with the termination of the sensitive period for odor attachment learning at PND10, concomitant with the functional emergence of amygdala activity to support odor aversion learning [61]. Indeed, infant rats after the sensitive period (*i.e.*, PND > 9) learn an amygdala dependent odor avoidance and display immobility/freezing behaviors in response to the presentation of an odor that has been paired with foot shocks [56,60,142]. A causal link between avoidance/fear learning and amygdala activity was demonstrated in these older pups through temporal suppression of the amygdala by Muscimol (a GABA_A_-receptor agonist) during the odor-shock conditioning, which completely blocked odor aversion/fear learning [55,82]. The ecological significance for the termination of the sensitive period for odor attachment learning may be related to the pups’ concomitant emergence of walking and the increased frequency of leaving the nest to explore its proximal environment. Perhaps at this age, a more contingency-based learning system emerges, which enables the preweanling rat to learn to avoid stimuli that are associated with danger outside the nest and prepare for independent life.

The ability of the amygdala to mediate fear learning after PND10 overlaps with the termination of the “stress hyporesponsive period” (SHRP), when infant rats show increased plasma CORT levels in response to various transient stress- and painful stimuli, as measured by radioimmunoassay of blood samples that have been collected either by decapitation or by jaw/tongue cannulations [147,148,149,150,151,152,153]. Additionally, previous research implicated a gradual increase of CORT levels in rat pups as important mediators for the emergence of unconditioned fear responses to naturally fearful odors, such as predator odors [154,155,156,157]. Similarly to conditioned fear, the occurrence of fear-related behaviors associated with predator odors emerge around PND10, which are accompanied by increased endogenous CORT levels [155]. Together, these results suggested that attenuated stress-induced CORT releases during odor-shock conditioning might be critically involved in the inability of pups to learn an aversion/fear towards a specific stimulus during the sensitive period. In addition, these results also indicate that CORT may serve as a causal mediator of switching the neural circuitry associated with attachment learning (engaging the olfactory bulb and the anterior piriform cortex) to those required for aversion/fear learning (engaging the amygdala and posterior piriform cortex). Indeed, during the sensitive period peripheral injections of CORT just prior to odor-shock conditioning engaged the amygdala and the posterior piriform cortex and allowed the expression of fear-related behaviors. Conversely, in older pups, the depletion of CORT, by adrenalectomy or by targeting its effects through systemic injection of the glucocorticoid receptor antagonist Mifepristone, blocked amygdala activity and the expression of fear and odor avoidance [81,158,159]. The property of the amygdala as the critical site for the permissive effects of CORT was further validated by intra-amygdala infusion of CORT receptor agonists and antagonists. While in pups below the age of 10 days, stimulation of amygdala CORT receptors during odor-shock conditioning prevented the attachment learning and induced an odor avoidance/fear response, odor avoidance/fear learning could be prevented in older pups (*i.e.*, PND > 9) by blockage of the amygdala glucocorticoid receptors . It is important to note that both mineralocorticoid and glucocorticoid receptors are already widely expressed throughout the brain [160,161], including the amygdala [162,163] by the first postnatal week. Interestingly, by PND16 this CORT controlled amygdala-dependent fear learning terminates and a more adult-like learning system emerge where CORT modulates learning and memory processes [116,158,164,165]. Taken together, these studies demonstrate that stress and its associated hormones (both CORT and NE) play a critical role in modulating the attachment learning system of rat pups during early development. 

## 5. Maternal Modulation of Pups’ Corticosterone Levels and Attachment Learning

At an age when rat pups show a pain-induced increase in circulating CORT levels (*i.e.*, > PND 9), the presence of the mother can prevent the shock-induced increase of CORT through “social buffering”, referring to a blunting of the hormonal stress response that occurs within a specific social context [151,152,166,167,168]. This suggested that, if increased CORT levels were required for amygdala-dependent odor aversion learning, then the mother could block odor aversion/fear learning in pups between PND10 and 15 (*i.e.*, after the sensitive period but before the infant amygdala loses its unique dependence on CORT to modulate odor attachment learning and amygdala plasticity). Indeed, when pups above the age of 9 days were odor-shock conditioned in the presence of an anesthetized mother an odor preference (instead of the typical odor aversion responses) was observed the following day, similarly to those found in pups during the sensitive period [82,169]. Strikingly, the ability of maternal presence to reinstate odor preference learning could be overridden with systemic or intra-amygdala administration of CORT just before odor-shock conditioning [82]. The ecological significance of the maternal presence in determining pups’ odor learning capabilities may be explained by developmental needs and changing environment of pups as they mature. For example, while neonatal pups during the sensitive period are confined to the nest and depend solely on the mother, older pups still depend on the mother for care but start to leave the nest and spend an increasing amount of time outside, to explore its proximal environment. Thus, during such a “Transitional Sensitive Period”, the presence of the mother enables pups to learn a preference for the diet-dependent maternal odor, which is needed for proximity seeking, nursing and nutrition, but at the same time, pups are able to learn to avoid threats outside the nest via access to the fear/avoidance learning system while away from the mother. Interestingly, such a social buffering of stress-induced CORT release occurs not only during early development but also throughout life in a vast number of mammalian species, including guinea pigs, voles, nonhuman primates and humans [170,171,172,173,174,175]. 

Additionally, the mother is able to increase pups’ CORT levels through her milk, when she is stressed [176], but also by sparse maternal care [167,177,178] or by frequent rough handling (maltreatment) of her pups [90,179,180]. The hormonal and neurobiological effects in pups reared by such a stressed and “maltreating” mother have recently been studied using a neonatal chronic stress paradigm, developed by the Baram lab [180,181,182]. In this neonatal stress paradigm rat mothers were stressed by providing them with insufficient bedding to build a nest, resulting in frequent nest building, trampling and rough handling of the pups as well as decreased nursing, which nevertheless manifested in none or only modest reduction in the pups body weight gain [90,179,181,183,184]. However, pups reared by such a stressed/maltreating mother during the sensitive period showed increased basal CORT levels [179,181] and disturbed social interaction with the mother, as indicated by reduced approach responses towards the maternal odor, as well as decreased time the pups spent nursing [90,183]. Since such maltreated pups have increased CORT levels and abnormal social behaviors, their odor attachment learning system was also disrupted. Specifically, when these maltreated pups were odor-shock conditioned at PND7, they learned an age-atypical odor aversion concomitant with a heightened amygdala activity, suggesting that frequent rough maternal care prematurely ends pups’ sensitive period for attachment learning [183]. Strikingly, treatment of these pups with systemic Mifepristone before odor-shock conditioning, which blocks the effects of conditioning-induced CORT release, reinstated odor preference learning, which further supported a causal involvement of CORT in terminating the sensitive period for attachment learning. 

It is important to note, that acute and prolonged maternal maltreatment has different behavioral and neurobiological outcomes. Whereas brief experience (*i.e.*, two 30min sessions) with a stressed/maltreating mother during the sensitive period does not substantially increase pup’ CORT levels and still produces attachment learning [60], prolonged (*i.e.*, 3 to 5 days) exposure to a maltreating mother during the same developmental period results in enduring elevation of circulating CORT levels and odor aversion/fear learning in response to odor-shock conditioning [90,179,180,182]. This suggests that during the sensitive period, pups can learn to prefer and approach a novel odor, associated with transient pain as long as the painful stimuli don’t involve heightened CORT levels, while a more frequent or prolonged pain and/or maltreatment and its induced increase in CORT levels result in odor avoidance learning. In general, while sensory stimulation provided by the mother during nursing and grooming suppresses pups’ CORT levels, prolonged maternal deprivation (few hours) or continuous experience with a maltreating mother (few days) are able to raise pups’ CORT levels for longer periods, resulting in a disrupted attachment with negative outcomes on brain and behaviors [149,151,184,185,186,187,188]. Taken together, these studies indicate that the mother, through maternal care, can critically influence pup’ CORT levels and thereby modulate attachment processes, which in turn may be critically involved in pre-programming the pup’s developmental trajectories. 

## 6. Enduring Effects of Early Life Stress and Attachment

Since the 1950’s, significant clinical and basic research has demonstrated that the developing brain is extraordinarily sensitive to environmental influences and that early-life experiences can exert profound effects on brain and behavior [189,190,191,192,193,194]. Original studies, as well as more recent work, have provided converging evidence that early-life experiences, such as quantity and quality of maternal care or manipulations of environmental variables such as handling, novelty exposure or the experience of various aversive stimuli can have profound effects on the individual’s developmental trajectories [9,45,151,194,195,196,197,198,199,200,201,202,203,204,205,206,207,208,209,210,211,212,213,214,215]. Strong evidence emerging from these studies point out that early life manipulations can exert enduring cognitive and emotional alterations, with CORT and disruption of the HPA axis identified as one potential mediator [215,216,217,218,219]. These sustained alterations of the HPA axis and its immediate and enduring effects on the brain have identified the important role of stress hormones in organizing the emotional and cognitive development. More recent research has unraveled some of the molecular mechanisms that underlie these long-lasting changes in stress responsivity by early life experiences, such as altered expression of hypothalamic corticotrophin releasing hormones (CRH) and of the glucocorticoid receptors in the hippocampus [9,213,220]. Importantly, the role of the stress system in mediating early life’s enduring effects has been reported in a variety of species, including rodents, humans and nonhuman primates, suggesting an evolutionary conserved mechanism that strengthens the link between clinical and basic research [200,221,222,223,224,225,226,227]. 

Strikingly, the repeated odor-shock conditioning of pups during the sensitive period, which results in a learned odor preference and produces odors that modulates pup’ attachment behaviors similarly to a new maternal odor, have also been reported to have enduring effects on brain and behaviors and may provide additional insight into mechanisms and outcome of early life traumatic experiences. However, the emotional outcomes of repeated odor-shock conditioning during infancy critically depend on the specific context in which the infant’s painful stimulus occurred. For instance, infant adverse experiences using electric foot shocks suggest, that predictable foot shocks produce different outcome on adult emotionality than unpredictable shock [10,189]. For instance, unpaired odor-shock conditioning in infancy produced increased anxiety-like behaviors in the dark-light emergence test in adulthood [228], whereas adults that experienced odors paired with foot shocks during infancy showed increased depressive-like behaviors in forced swim, sucrose preference and social behavior tests [95]. Potential explanations for this difference on the emotional outcome in adulthood between rat pups that received predictable versus unpredictable footshocks are differences in the release and/or turnover of NE in different brain regions (*i.e.*, hypothalamus, amygdala and locus coeruleus), similarly to those that have been reported in adult rats in response to either predictable or unpredictable stressors [229,230]. Interestingly, these depressive-like behaviors of adult rats, that received paired odor-shock conditioning during infancy, were also accompanied by disrupted amygdala activity [95]. Importantly, both depressive-like behaviors in the forced swim and social behavior tests as well as amygdala dysfunctions could be brought to control levels by simply presenting the odor that was initially paired with foot shocks during infancy [231]. Additional work suggests also that such odors used in odor-shock conditioning during infancy can also attenuate fear learning and amygdala activity in adult animals [93,94]. Although these findings imply that odors acquired during early infancy can attenuate depressive-like behaviors in adult rats, it remains to be investigated whether similar “anti-depressant”-like properties of early life odors (or other aspects of the early life environment) also exist in other altricial species, including humans. Together, these results suggest that odors paired with shocks during the sensitive period for attachment learning in infancy take on characteristics of the maternal odor, and continue to have powerful control over emotions and stress responses later in life. 

## 7. Summary and Conclusions

In neonatal rats, odors mediate the infants’ attachment to their mother. These odors are acquired both pre- and postnatally and are encoded by a unique neural circuit that involves a hyper-functioning of the locus coeruleus in response to a broadly categorized reward system (including painful stimuli) to induce odor approach learning. This system also requires hypo-functioning of the amygdala to suppress odor avoidance/fear learning when odors are associated with painful stimuli. While this strong and robust odor approach learning of neonatal rat pups ensures attachment to the caregiver, which is necessary to signal needs and obtain parental care, the pup’ limitations of aversion learning during the sensitive period might be equally important for ensuring optimal approach responses and social interactions with the caregiver. Changing activity of the locus coeruleus in response to sensory stimuli together with the functional interaction between the CORT system, the amygdala and the learning context (*i.e.*, whether the mother is present or absent) are critically involved in the developmental switch from odor approach to odor avoidance/fear learning and enable the infant’s attachment system to be optimally adapted to its relative ecological niche. Frequent external stress and/or an abnormal early social environment during the sensitive period for attachment learning critically alters this finely tuned system, resulting in both short- and long-term consequences on brain and behavior. In particular elevated levels of CORT can critically disturb the mother-infant interactions and thereby appears to exert negative influences on the infant’s development, which may be partially responsible for the enduring effects of early life stress.

Together, this rodent model of attachment, when combined with other rodent models of early life experience, enables the study of specific components and perturbations of the early social environment and provide powerful tools for a more comprehensive understanding of the neurobiological mechanisms of how early life stress interacts with other neural systems and thereby modulates ecologically relevant behaviors. 
